# Individually Cultured Bovine Zygotes Successfully Develop to the Blastocyst Stage in an Extremely Confined Environment

**DOI:** 10.3390/cells13100868

**Published:** 2024-05-17

**Authors:** Angela Travaglione, Andrea Candela, Vincenza De Gregorio, Vincenzo Genovese, Mario Cimmino, Vincenza Barbato, Riccardo Talevi, Roberto Gualtieri

**Affiliations:** Department of Biology, University of Naples “Federico II”, Complesso Universitario Di Monte S. Angelo, Via Cinthia, 80126 Naples, Italy; angela.travaglione@unina.it (A.T.); andrea.candela@unina.it (A.C.); vincenza.degregorio@unina.it (V.D.G.); vincenzo.genovese@unina.it (V.G.); mariocimmino1927@gmail.com (M.C.); barbato_vincenza@libero.it (V.B.); riccardo.talevi@unina.it (R.T.)

**Keywords:** individual embryo culture, confined microenvironment, group embryo culture, blastocysts developmental competence

## Abstract

The possibility of detecting the developmental competence of individually cultured embryos through analysis of spent media is a major current trend in an ART setting. However, individual embryo culture is detrimental compared with high-density group culture due to the reduced concentration of putative embryotropins. The main aim of this study was to identify an individual culture system that is not detrimental over high-density group culture in the bovine model. Blastocyst rates and competence were investigated in a conventional (GC) group, semi-confined group (MG), and individual culture (MS) in a commercial microwell device. Main findings showed that: (1) individual embryos can be continuously cultured for 7 days in ~70 nL microwells (MS) without detrimental effects compared with the GC and MG; (2) MS and MG blastocysts had a reduced number of TUNEL-positive cells compared to GC blastocysts; (3) though blastocyst mean cell numbers, mitochondrial activity, and lipid content were not different among the three culture conditions, MS blastocysts had a higher frequency of small-sized lipid droplets and a reduced mean droplet diameter compared with GC and MG blastocysts. Overall, findings open the way to optimize the development and competence of single embryos in an ART setting.

## 1. Introduction

Despite the continuous development of new procedures for selecting euploid and competent embryos to transfer in human assisted reproductive technologies (ART), only a few studies have been focused on biophysical aspects of current in vitro preimplantation embryo culture procedures, which has remained essentially unchanged over the years as we had already reached the maximum levels of efficiency [1,2,3]. However, studies in animal models and domestic species clearly indicate that in vitro-produced (IVP) embryos have a lower developmental competence than their in vivo-derived (IVD) counterparts, in terms of blastocyst rates, euploidy, cryotolerance, gene expression, and pregnancy rates [4,5,6,7,8,9,10,11]. In vivo, embryos develop within confined microenvironments—the oviduct (or fallopian tube in humans), and the uterus—virtual cavities where highly concentrated maternal and embryo-derived factors, in the form of proteins and/or other molecular cargos enclosed within extracellular vesicles, promote preimplantation embryo development and affect embryo developmental competence [12,13,14,15,16]. Although the bidirectional crosstalk between the embryo and the maternal reproductive tract is obviously absent during in vitro embryo culture, evidence indicates that the potential benefits of autocrine embryo secretions, the so-called embryotropins, could depend on the specific culture system adopted [17]. In vitro embryo development is ameliorated when multiple embryos are cultured in a certain volume of culture medium compared to a single embryo cultured in the same volume. Such an improvement, known as a “group effect”, has been suggested to arise from the increased concentrations of embryotropins during group embryo culture [12,13]. According to these concepts, different studies were addressed to improve the in vitro embryo culture conditions, reducing the volume of the culture medium drops under mineral oil [18,19]. However, reduced drop volumes exerted detrimental impacts on bovine embryo development [20], likely due to the increased surface exposed to oil relative to the drop volume, resulting in a higher in and out exchange of apolar solutes between the oil and medium [21,22,23,24,25,26,27]. Vajta et al. (2021) introduced a semi-confined group culture procedure, termed “Well of the Well” (WOW), whereby multiple embryos individually reside within microwells while sharing a common drop of medium with the other embryos (reviewed in [1]). This approach should maintain the benefits of group culture through the establishment of a semi-confined microenvironment in which positive-effects autocrine secretions are enriched, while the common drop of medium prevents the depletion of nutrients and the accumulation of waste metabolites [1,28,29]. Different microwell devices have been largely adopted in human ART to allow for the collection of morpho-kinetic parameters of individual embryos, though the actual advantage of embryo morphokinetics for the selection of high-quality embryos remains unclear [30,31]. The current trend in human ART is to select and transfer a single euploid and competent blastocyst through the application of novel non-invasive embryo quality markers [32]. “Omics analysis”, i.e., the determination of metabolome, transcriptome, secretome, genome, etc., of single embryos, studying the spent culture media of individual embryo cultures to evaluate the euploidy and developmental competence of embryos to transfer could soon become a reality [33]. Under this scenario, the development of individual embryo culture systems without detrimental effects compared to group culture becomes mandatory to ensure an actual embryo’s individuality and allow for the application of these methods. As obvious ethical reasons prevent direct experimentation on humans, in the present study, the bovine was selected as an animal model for its closer similarity to human embryo development compared to the mouse model [34,35]. In this perspective, we established an individual embryo culture that maintains quality and viability comparable to conventional group culture, mimicking the reduced volume experienced by embryos in the oviductal tract. 

## 2. Materials and Methods

### 2.1. Oocyte Retrieval and In Vitro Maturation

Bovine ovaries were obtained from Di Tella S.R.L. slaughterhouse (San Marcellino, Caserta, Italy; CEE accreditation number 1403/M) and transported to the laboratory within 3 h at 33 °C. Ovaries were washed with pre-warmed physiological saline supplemented with 100 IU Penicillin and 100 µg/mL streptomycin, and cumulus oocyte complexes (COCs) were collected into 15 mL conical-bottom tubes (Falcon; Milan, Italy) containing 70 µL of heparin 10 mg/mL through aspiration from antral follicles (2–8 mm diameter) using an 18-gauge needle. COCs with a compact, at least three-layered non-atretic cumulus, and an oocyte with homogeneous cytoplasm were selected, washed in wash medium (Stroebechmedia, Copenhagen, Denmark), and matured for 22–24 h in groups of 50 in four-well dishes (Nunc—Termo Fischer Scientific, Roskilde, Denmark) containing 500 µL of IVM medium (Stroebechmedia, Copenhagen, Denmark) at 38.5 °C, 6% CO_2_ in air, 95% humidity. 

### 2.2. Semen Preparation, In Vitro Fertilization and Embryo Culture

Frozen bovine semen from three bulls (0.5 mL straws—approximately 15–20 × 10^6^ spermatozoa per straw—motility after thawing >70%) was obtained from Intermizoo S.p.a (Padova, Italy). Straws were thawed in a water bath at 37 °C for 1 min, and semen was rinsed in 10 mL of semen wash medium (Stroebechmedia, Copenhagen, Denmark) by centrifugation at 170× *g* for 10 min. The sperm pellet was resuspended in 700 μL of IVF medium, sperm concentration was determined with a Burker chamber, and sperm motility was evaluated by a Sperm Class Analyzer (SCA, Microptic S.L., Barcelona, Spain) using a Makler chamber placed on a microscope stage at 38 °C. Sperm motility was analyzed at a Nikon TE 2000 (Nikon, Tokio, Japan) inverted microscope connected to a Basler Vision Technology A312 FC camera (Basler, Ahrensburg, Germany) with a positive-phase contrast 10× objective. At least 200 cells and four/five fields were acquired and analyzed for each sperm suspension. Progressive motility and kinetics were evaluated by SCA in terms of curvilinear velocity (VCL), straight-line velocity (VSL), and average-path velocity (VAP). The following software settings were used: 25 frames/s, 10 frames/object, 10 µm/s velocity limit for slow sperm, 25 µm/s velocity limit for average sperm, 50 µm/s velocity limit for rapid sperm, 50% minimal linearity, and 70% straightness for progressive fast sperm. For in vitro fertilization, 45–50 matured COCs for each well were suspended in 250 µL IVF Medium (Stroebechmedia, Copenhagen, Denmark), inseminated with 250 µL of sperm suspension (2 × 10^6^/mL—final sperm concentration), and co-incubated for 19–22 h at 38.5 °C, 6% CO_2_ in air, 95% humidity. At 19–22 h post insemination (p.i.), the presumptive zygotes (*n* = 1073) were denuded by vortexing (2 mL volume, max velocity for 3 min) and then cultured under the different culture conditions, as reported below, in IVC medium (Stroebechmedia, Copenhagen, Denmark) for 7 days at 38.5 °C, 6% CO_2_, 6% O_2_ and 88% N_2_, 95% humidity. At day 8 p.i., blastocyst rates and stages were assessed, and blastocysts were treated for determination of cell numbers, TUNEL-positive cells, mitochondrial activity, and lipid content, as detailed below.

### 2.3. Culture Devices and Loading of Embryos or Presumptive Zygotes

**Figure 1:**
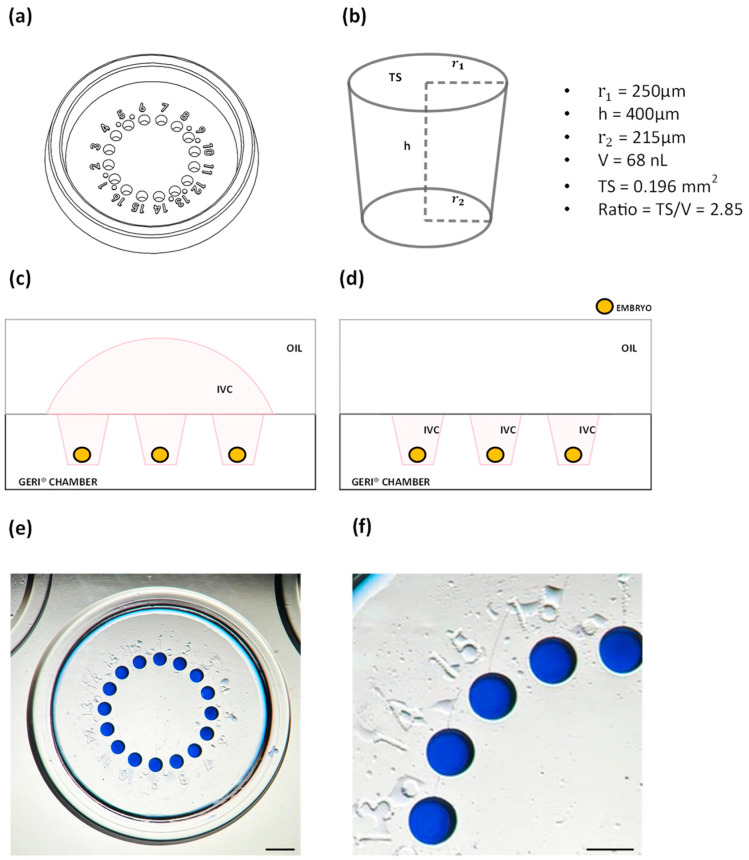
Drawings of the WOW-based GERI^®^ chamber (**a**–**d**) and representative images of MS dye loading experiment to verify medium communication between adjacent microwells (**e**,**f**). (**a**) Schematic drawing of GERI^®^ chamber main well. (**b**) Dimensions, volume and surface/volume ratio of an individual microwell. TS = top surface; ratio = ratio of surface exposed to oil (TS) and microwell volume (V). (**c**,**d**) Drawings of MG (**c**) and MS (**d**) culture conditions. IVC = embryo culture medium. (**e**,**f**) Representative images of dye-loaded MS after 7 days incubation (**e**) showing lack of communication (**f**) between adjacent microwells. Bar = 1 mm (**e**); bar = 0.5 mm (**f**).

Each experiment was performed using commercial polystyrene microwell chambers (GERI^®^ chambers—GENEA BIOMEDX, Sidney, Australia) and NUNC™ four-well dishes. The GERI^®^ chamber consists of 4 wells, one of which includes 16 contiguous microwells that allow for the spatial confinement of single embryos (Figure 1a). Each microwell has an inverted truncated cone shape, with a base diameter of 430 µm, an upper diameter of 500 µm, and a height of 400 µm. Therefore, each microwell has a volume of 68 nL and a surface (exposed to oil)/volume ratio of 2.85 (Figure 1b). GERI^®^ chambers were loaded following two different procedures: (i) microwell group culture in a semi-confined environment (MG), in which 16 embryos (or presumptive zygotes) were loaded into microwells in a shared 60 µL medium drop (embryo density 1/3.75 µL) and then covered by 3 mL of mineral oil (Strobechmedia, Copenhagen, Denmark) (Figure 1c); (ii) microwell single culture in a confined environment (MS), in which 16 embryos (or presumptive zygotes) were loaded as in MG and then the shared 60 µL drop of medium was removed through two repeated 60 µL aspirations with the pipette tip vertically positioned at the center of microwell array (embryo density 1/68 nL) (Figure 1d). In all experiments, conventional group embryo culture (GC), consisting of groups of 50 presumptive zygotes/500 µL per well (embryo density 1/10 µL), served as control.

Preliminary dye loading experiments were performed to demonstrate the lack of medium communication between adjacent microwells during individual culture under the MS condition. To this end, in a first series of experiments, after MS loading, a few nL of toluidine blue 1% was gently injected into microwells through a Cook^®^ Flexipet^®^ Pipette (Cook, Milan, Italy). Micrographs of dye-loaded MS were acquired before and after incubation for 7 days. At the end of culture, diffusion of the dye from a dye-loaded microwell to the adjacent non-dye-loaded microwell was seldom present. However, this dye loading procedure did not faithfully reflect the MS embryo loading procedure as an additional volume of stain was injected into the microwells. For this reason, in a second series of experiments, toluidine blue was directly dissolved in IVC medium, microwell chambers were loaded according to the MS procedure (Figure 1e), and micrographs were acquired before and after 7 days incubation (Figure 1f) to verify the possible presence of residual films of dye putting adjacent microwells in communication. Findings showed the lack of residual films of culture medium putting adjacent microwells in communication, demonstrating that embryos are individually cultured under this procedure.

### 2.4. Experimental Design

*Experiment 1*: The first series of experiments (*n* = 5; total oocytes 673) was addressed to investigate the blastocyst rates and quality obtained after 3 days of culture in conventional group culture (GC) followed by culture for a further 5 days in semi-confined (MG) or confined (MS) environment. To this end, embryos cultured 3 days p.i. in GC were left undisturbed or randomly allocated in MG and MS and cultured until day 8 p.i. in 6% CO_2_, 6% O_2_ and 88% N_2_, with 95% humidity at 38.5 °C. Blastocyst rates and stages were recorded at day 8 p.i. Blastocyst cell numbers and percentage of TUNEL-positive cells were determined as described below.

*Experiment 2*: Results of experiment 1 indicated that day 3 p.i. embryos transferred to MG and MS were able to develop until the blastocyst stage but with reduced rates compared to conventional GC. Therefore, in experiment 2, presumptive zygotes at day 1 p.i. (*n* = 6; presumptive zygotes 776) were loaded after removal of cumulus cells in GC, MG, and MS, cultured until day 8 p.i. and analyzed as described in experiment 1.

**Figure 2:**
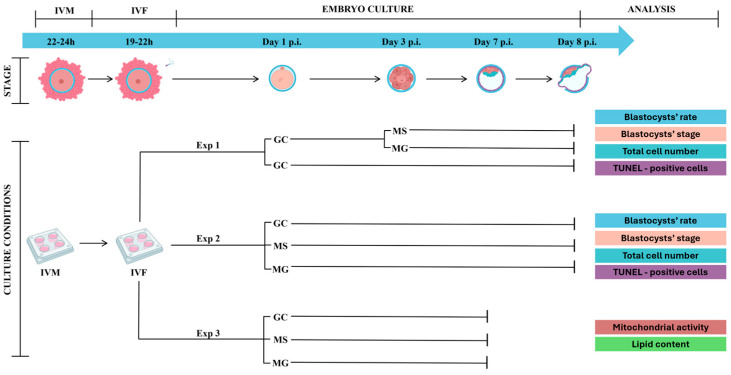
Graphic experimental design. GC = group culture; MG = microwell group culture; MS = microwell single culture; p.i. = post insemination. Figure created with BioRender.com (accessed on 11 April 2024).

*Experiment 3*: Day 7 blastocysts produced in GC, MG, and MS (*n* = 3; 60 blastocysts) as described in experiment 2, were treated for determination of mitochondrial activity and lipid content as indicators of developmental competence, as detailed below (Figure 2). 

### 2.5. Embryo Development and Quality Assessment

For all culture conditions, cumulative blastocyst yields were recorded at day 8 p.i. under a Nikon SMZ18 (Nikon, Florence, Italy). Blastocysts rates were assessed and expressed as percentages of blastocysts on cleaved embryos. Blastocyst developmental stages were expressed as percentages of early-expanded blastocysts and hatching/hatched blastocysts on total blastocysts. 

#### 2.5.1. *TUNEL Assay*

The TUNEL assay was used to assess DNA fragmentation in blastocysts (*n* = 167). The terminal free 3′-OH ends of DNA were labelled with dUTP conjugated to red fluorescent dye tetramethylrhodamine (In Situ Cell Death Detection Kit, TMR red, Merck, Milan, Italy) using terminal deoxynucleotidyl transferase. At day 8 p.i., blastocysts from GC, MG, and MS cultures were fixed in 4% paraformaldehyde (PFA, Sigma Aldrich, Milan, Italy) in PBS overnight at 4 °C. After three 5 min washings in PBS with 0.1% polyvinylpyrrolidone (Sigma Aldrich, Milan, Italy) (PBS-PVP), blastocysts were permeabilized in 0.1% Triton X-100 (Sigma Aldrich, Milan, Italy), 0.1% sodium citrate (Sigma Aldrich, Milan, Italy) for 30 min at 4 °C, washed three times for 5 min in PBS-PVP, and then incubated for 1 h in the TUNEL reaction mixture at 37 °C in the dark according to the manufacturer’s instructions. Blastocysts were then washed in PBS-PVP, labelled with 10 µg/mL Hoechst 33342 (Sigma Aldrich, Milan, Italy) for 7 min at room temperature (r.t.), washed again, mounted on a microscope slide in 10 µL of PBS-PVP, covered with 24 × 24 mm coverslips, and sealed. Images were acquired at a Nikon Eclipse TE2000-U connected to a Nikon DS-5Mc video camera with a 40×, 1.3 N.A. objective through NIS-element BR 4.6 software (Nikon, Florence, Italy). Hoechst-stained nuclei were visualized through a UV filter (λex 350 nm, λem 461 nm), and TUNEL-positive nuclei through a TRITC filter (λex 544 nm, λem 570 nm; Nikon, Florence, Italy). Negative controls were prepared omitting terminal deoxynucleotidyl transferase in the reaction mixture, while positive controls were prepared through pretreatment with 1 mg/mL DNase I for 10 min at r.t. Blastocyst total cell and TUNEL-positive cell numbers were quantified using the ‘total cell counter’ of Image J 1.54f software (NIH, New York, NY, USA).

#### 2.5.2. *Blastocyst Mitochondrial Activity and Lipid Content*

*Blastocyst staining*: Day 7 blastocysts (*n* = 60; 20 for each culture condition) were assessed for mitochondrial activity and lipid content. Blastocysts from each culture condition were washed twice in IVC medium, incubated for 30 min at 38.5 °C in 400 nM MitoTracker DeepRed (Molecular Probes, Eugene, OR, USA) in IVC medium, washed 3 × 5 min in PBS-PVP, and then fixed in 4% PFA 30 min at r.t. Fixed blastocysts were permeabilized in 0.1% saponin in PBS for 30 min and stained 1 h at r.t. in 20 μg/mL Bodipy (493/503, Thermofisher Scientific, Parma, Italy) in PBS-PVP. After 3 × 5 min washings in PBS-PVP, the blastocysts were stained for 30 min in 10 μg/mL Hoechst 33342 in PBS-PVP, washed 3 × 5 min in PBS-PVP, mounted on microscope slides in 50% glycerol-PBS, covered with round coverglass with spacers to retain the blastocyst three-dimensional shape, and sealed. Blastocysts were imaged with a 60× oil immersion objective at a FV3000 OLYMPUS laser-scanning confocal microscope (Olympus Italia, Segrate, Italy) equipped with a 488 nm argon laser (emission 500–540 nm) for visualization of lipid droplets, a 640 nm laser (emission 650–750 nm) for visualization of mitochondria. 

*Assessment of mitochondrial activity:* total fluorescence signal intensity was quantified as follows: 8–12 3 μm serial optical sections and a maximal projection were acquired for each blastocyst. Image analysis was performed using ImageJ software (NIH; ImageJ version v1.54i software). Following selection through the freehand selection tool, each blastocyst maximal projection was measured to determine its area and its integrated density (IntDen), corresponding to pixel intensity. An area outside the blastocyst was measured for obtaining the background fluorescence. Fluorescence intensity in each blastocyst was determined as follows: relative fluorescence = IntDen − (area of selected blastocyst × mean background fluorescence). Fluorescence intensities were expressed in arbitrary units (a.u.). 

*Assessment of lipid content:* Blastocysts lipid quantification was performed by analyzing the total area of lipids in each blastocyst. Three 1024 × 1024 images were captured for each blastocyst: one equatorial and two in the middle of the resulting halves. Images were analyzed using the ‘nucleus counter’ tool, set to quantify droplet areas with the ImageJ software. Lipid quantity was corrected by the total embryo area to account for varying blastocyst sizes [36]. In addition, an analysis for lipid droplets (LDs) in terms of size frequency was conducted on the same blastocysts previously examined. A series of 8-bit z-stack images were captured for each embryo at 3 μm intervals, starting from the top slice (containing the first LDs capture) to the final slice. The acquired optical sections were analyzed through the Image J 1.54f software (NIH, USA). LDs parameters were measured through the following steps: background subtraction (rolling ball radius = 10), threshold adjustment (55–255), binary image conversion, watershed transformation (to remove the potential LDs clusters), and analyzing particles tool (excluding particles with the mean area <0.07 µm^2^ to avoid counting background noise). Additionally, an Excel file was created to categorize LDs size frequency into five classes: 0.07–0.3, 0.3–5, 5–15, 15–30, and >30 µm^2^. For all analyzed parameters, the value of each slice was calculated separately, and the mean value per slice was determined. 

### 2.6. Statistical Analysis 

Each experiment was conducted at least in biological triplicate, and the results were presented as mean ± standard deviation (SD). Statistical analyses were performed using GraphPad Prism Software (version 8.02 for Windows, GraphPad Software, Boston, MA, USA). For the analysis of mitochondrial activity and LDs parameters, one-way analysis of variance (ANOVA) was employed, followed by Tukey’s pairwise comparison tests. Blastocyst rates, stages, total cell numbers, and TUNEL-positive cells were expressed as cumulative percentages and analyzed using Fisher’s exact test for pairwise comparisons when an overall significance was observed. *p* < 0.05 was considered statistically significant. 

## 3. Results

**Figure 3:**
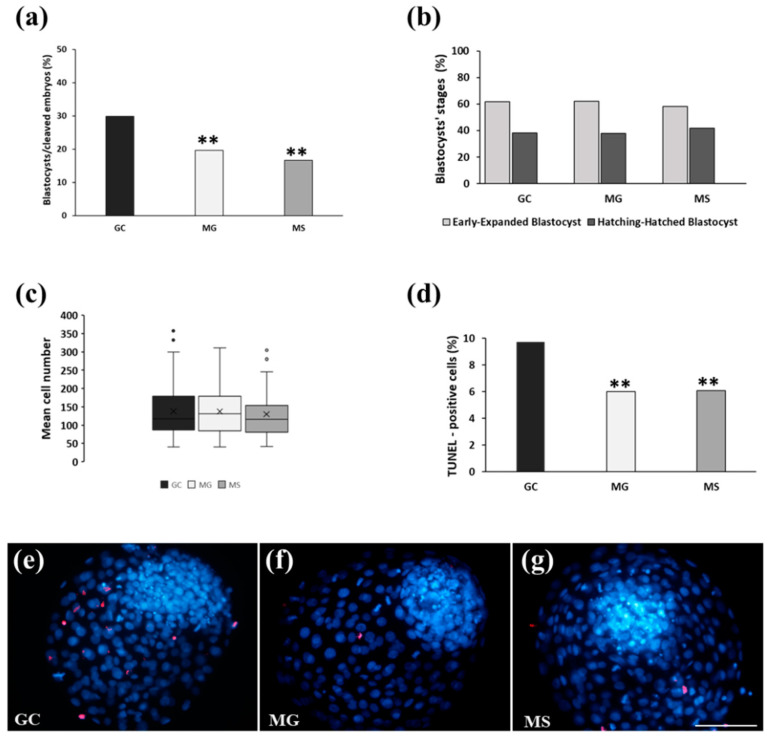
Experiment 1: Uninterrupted culture in GC compared with culture in GC until day 3 p.i. followed by MG and MS culture until day 8 p.i. (**a**) Blastocyst’s rates. (**b**) Blastocyst’s stages. (**c**) Blastocyst’s mean cell numbers. (**d**) Percentages of TUNEL-positive cells. (**e**–**g**) Representative images of cell numbers (blue) and TUNEL-positive cells (red) in blastocysts cultured in GC, MG and MS. Bar = 100 µm. **, *p* < 0.01 vs. GC.

*Experiment 1*: In the first series of experiments, the cleavage rate at day 3 p.i. was 88.8%. Blastocyst’s rate at day 8 p.i. (Figure 3a) in GC was markedly higher than that in MG and MS (29.9 vs. 19.6 and 16.7%; *p* < 0.001). No differences were observed among the three culture conditions in terms of blastocyst’s stages (Figure 3b). Blastocyst cell numbers and percentages of TUNEL-positive cells were assessed as markers of developmental competence. The mean blastocyst’s cell number was not significantly different among the three culture conditions (GC, 137.8 ± 71.7; MG, 137.3 ± 67.2; MS, 129.5 ± 59.9) (Figure 3c). Conversely, the percentage of TUNEL-positive cells cultured in GC was significantly higher compared to that of both MG and MS blastocysts (9.7 vs. 6 and 6.1%; *p* < 0.01) (Figure 3d). Figure 3 shows representative images of blastocysts cultured in GC (Figure 3e), MG (Figure 3f), and MS (Figure 3g) stained with Hoechst 33342 to visualize cell nuclei (blue) and TUNEL to visualize DNA-fragmented cells (red). 

**Figure 4:**
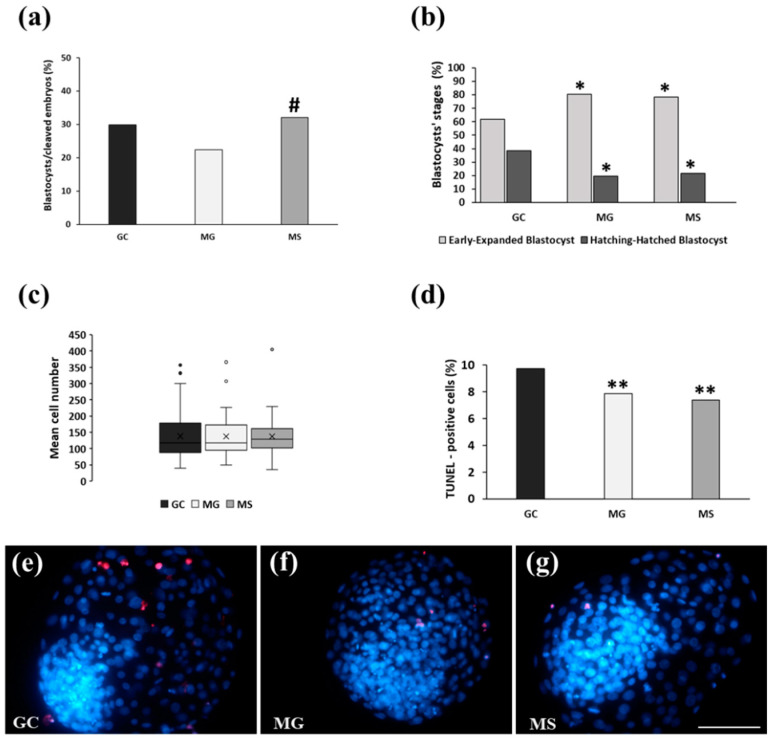
Experiment 2: Culture of presumptive zygotes from day 1 to day 8 p.i. in GC, MG and MS. (**a**) Blastocyst’s rates. (**b**) Blastocyst’s stages. (**c**) Blastocyst’s mean cell numbers. (**d**) Percentages of TUNEL-positive cells. (**e**–**g**) Representative images of cell numbers (blue) and TUNEL-positive cells (red) in blastocysts cultured in GC, MG and MS. Bar = 100 µm. #, *p* < 0.05 vs. MG; *, *p* < 0.05 vs. GC; **, *p* < 0.01 vs. GC.

*Experiment 2*: The findings of Experiment 1 showed a reduced blastocyst’s rate in embryos cultured in MG and MS from day 3 to day 8 p.i. compared with those uninterruptedly cultured in GC. In Experiment 2, presumptive zygotes were uninterruptedly cultured under the three conditions from day 1 to day 8 p.i. The following cleavage rates were detected at day 3 p.i. under the three embryo culture conditions: GC, 86.6%; MG, 83.8%; MS, 84.9%. Interestingly, blastocysts rate in MS (35.8%) was similar to GC (28.9%) and significantly higher than MG (23.1%; *p* < 0.05) (Figure 4a). No significant differences in blastocyst’s stages (Figure 4b) and mean cell numbers (Figure 4c) were detected among the three culture conditions (GC, 137.8 ± 71.7; MG, 137 ± 75.3; MS, 137.1 ± 60.3). Conversely, the percentages of TUNEL-positive cells in blastocysts cultured in MG and MS were significantly lower than those in GC (7.9 vs. 7.4 and 9.7%; *p* < 0.01) (Figure 4d). Figure 4 shows representative images of blastocysts cultured in GC (Figure 4e), MG (Figure 4f), and MS (Figure 4g), stained with Hoechst 33342 to visualize cell nuclei (blue) and TUNEL to visualize DNA-fragmented cells (red). 

**Figure 5:**
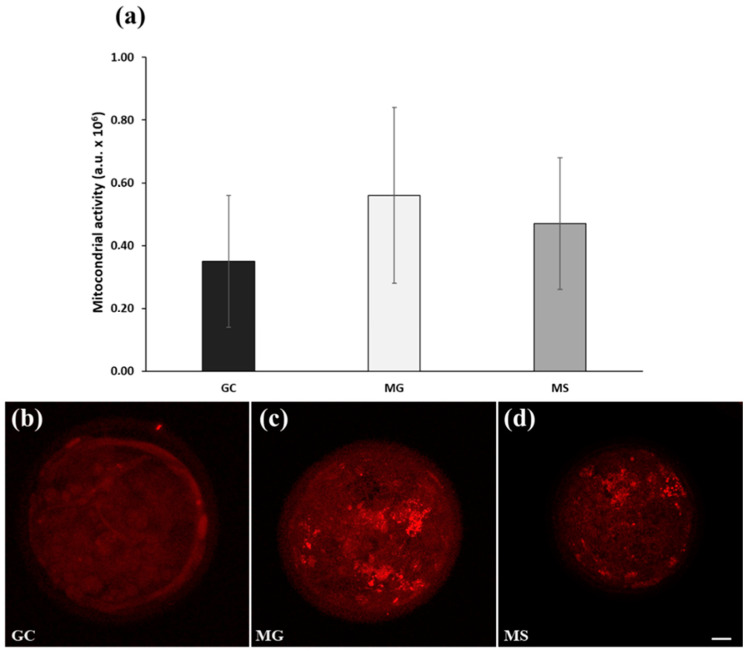
Experiment 3: Culture of presumptive zygotes from day 1 to day 7 p.i. in GC, MG and MS. (**a**) Mitochondrial activity expressed in arbitrary units (a.u.). (**b**–**d**) Representative images of mitochondrial activity in blastocysts cultured in GC, MG and MS and labeled in vivo with MitoTracker DeepRed. Bar = 20 µm.

**Figure 6:**
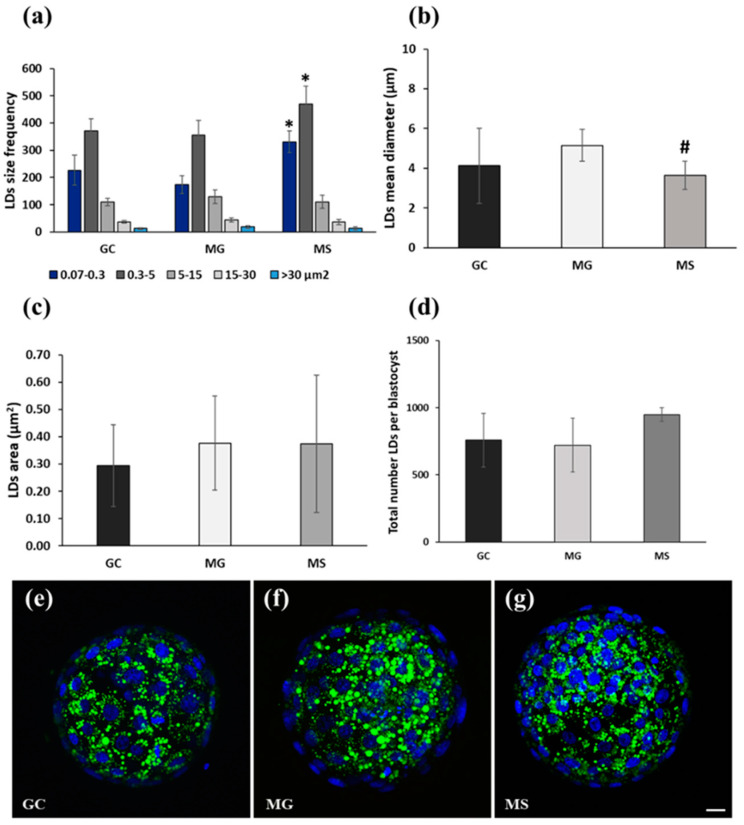
Experiment 3: Culture of presumptive zygotes from day 1 to day 7 p.i. in GC, MG and MS. (**a**) LDs size frequency expressed in µm^2^. (**b**) Mean diameter of LDs. (**c**) Quantification of the total area of lipid droplets (μm^2^). (**d**) Total number of LDs per blastocyst. (**e**–**g**) Representative images of cell numbers (blue) and Bodipy–stained LDs (green) in blastocysts cultured in GC, MG and MS. *, *p* < 0.05 vs. GC and MG; #, *p* < 0.05 vs. MG. Bar = 20 µm.

*Experiment 3*: Presumptive zygotes were cultured under the same conditions described in Experiment 2. The following cleavage rates were detected at day 3 p.i. under the three embryo culture conditions: GC, 81.7%; MG, 87%; MS, 87%. Mitochondrial activity and lipid analysis (LDs area; LDs size frequency) of day 7 blastocysts were evaluated as additional markers of developmental competence. Although not statistically significant, the mitochondrial activity was higher in MG and MS compared to GC blastocysts (Figure 5a). Figure 5 shows representative images of blastocysts cultured in GC (Figure 5b), MG (Figure 5c), and MS (Figure 5d), labeled in vivo with MitoTracker DeepRed to visualize mitochondrial activity. LDs size frequency quantification showed a significantly higher number of classes in a range 0.07–0.3 and 0.3–5 µm^2^ and a lower mean diameter (1.8 ± 0.48 µm) in MS compared to MG (2.53 ± 0.10 µm) and GC (2.28 ± 0.43 µm) conditions (Figure 6a,b). Blastocyst’s lipid contents were comparable among the three culture conditions (GC, 0.29 ± 0.15; MG, 0.38 ± 0.17; MS, 0.37 ± 0.25 µm^2^) (Figure 6c). Furthermore, the total number of LDs per blastocyst was slightly higher in MS (949 ± 50.18) compared to GC and MG (757.81 ± 200.15; 720.43 ± 200.37) (Figure 6d) conditions, although not statistically significant. Figure 6 depicts representative 3D reconstruction images of blastocysts cultured in GC (Figure 6e), MG (Figure 6f), and MS (Figure 6g), stained with Hoechst 33342 to visualize cell nuclei (blue) and BODIPY to visualize LDs (green). It was notable that in MS, a greater abundance of smaller size LDs occurred compared to GC and MG conditions (Figure 6e–g).

## 4. Discussion

The possibility of selecting competent blastocysts to transfer through omics assessment on spent culture media is a current trend in assisted reproduction both in human and domestic animal species [37,38,39]. However, such a procedure depends on the possibility of efficiently culturing individual embryos. Individual embryo culture negatively affects the ability to reach the blastocyst stage and blastocyst developmental competence compared with high-density group embryo cultures [17,40,41,42,43,44,45]. Thus far, the “group effect” has been clearly demonstrated in a number of poli- and mono-ovulatory species, including humans, (though controversial data were reported on the latter species) [12,13,44,46,47,48]. The lack of efficiency of individual versus group cultures depends on a number of factors, the dilution of embryo-secreted positive effects embryotropins being the most unfavorable condition [18,19]. Attempts to increase the concentration of such positive factors through a reduction in the volume of single embryo culture drops under oil must cope with the enhanced unwanted bidirectional apolar solute exchanges between oil and medium due to the increase in the drop surface relative to its volume and the possible deprivation of nutrients and accumulation of embryo-damaging waste metabolites [20,21,24,49,50]. Under this context, the most striking finding of the present study is the ability of bovine zygotes to be individually and continuously cultured for 7 days until the blastocyst stage in only ~70 nL of IVC medium without any detrimental effect in terms of mean cells numbers, mitochondrial activity and lipid content compared to standard group culture. To our knowledge, this is the first proof that such an extremely confined, static individual culture has an efficiency similar to high-density standard group culture. The minimal medium volume required to continuously culture a bovine embryo from day 1 to day 8 p.i. is thought to be a compromise among nutrient deprivation, waste metabolites accumulation, and putative embryotropins concentrations. In the case of culture in drops under oil, a further constraint of minimal required volume for individual embryo cultures is represented by the enhanced drop surface exposed to oil relative to the drop volume associated with its reduction. Such a constraint likely derives from the enhanced in and out unwanted solute exchanges between oil and medium when drop volume is progressively reduced. Oil has been shown to sequester from the culture medium apolar solutes such as steroid hormones and release toxic solutes like alkenals, aldehydes, triton X-100, etc., damaging embryo development [21,22,23,24,25,26,27]. This concept seems to be in contrast with the present findings, as the calculated surface/volume ratio during single microwell culture is 2.85, i.e., approximately equal to the ratio of 2.5 µL drops (approximating the drop geometry to a hemisphere) in which bovine zygotes reportedly do not successfully develop to the blastocyst stage. Carolan et al. (1996) cultured bovine embryos individually in droplets of 1, 2, 5, 10, or 20 µL overlaid with mineral oil and showed that embryo development was seriously compromised in droplet sizes of less than 10 µL. Moreover, individual culture in 10 and 20 µL droplets produced blastocysts with lower cell numbers and hatching rates compared with group cultures [20]. Similar findings were reported by O’ Doherty et al. (1997), showing detrimental effects on blastocyst rates when embryos were individually cultured in 10 µL drops compared to high-density group culture [51]. Conversely, our main findings demonstrate that in individual cultures, approximately 70 nL is enough to allow for blastocyst development. Compared with individual cultures in 10 µL drops, the putative embryotropins concentration achieved in ~70 nL of culture medium should be more than 140-fold higher. It can be speculated that such a higher concentration of positive-effect embryo secreted factors counterbalances the well-known detrimental effects of oil overlay at the same surface/volume ratio. This is in agreement with studies demonstrating the feasibility of extremely confined culture, using the microcapillary “Glass Oviduct” system (GO) in which oil is only in contact the two ends of the capillary, achieving an extremely low surface/volume ratio. GO systems support single bovine embryo development until the blastocyst stage for 7 days of uninterrupted culture in 1 μL [52,53]. In the present study, individual culture (MS) was compared with standard group culture (GC) at a density of 1 embryo/10 µL and semi-confined group culture (MG) in a microwell chamber at 1 embryo/3.75 µL. Blastocyst rates in MS were not different from those achieved in GC and were significantly higher than those achieved in MG. The mean cell numbers were not different among the three culture conditions, whereas the proportion of apoptotic cells in MS and MG was reduced compared to GC. Similarly, Sugimura et al. (2010) showed a reduced apoptosis in microwell semi-confined group culture compared to standard group culture at a density of 1 embryo/5 µL, while blastocyst morphological quality and inner cell mass and trophectoderm cell numbers were similar under the two culture conditions [54]. In the first experiment of our study, embryos were initially cultured in GC and then transferred to MG and MS at day 3 p.i. Although the mean cell numbers in blastocysts were consistent across all embryo culture conditions, those developed in MG and MS exhibited significantly fewer apoptotic cells compared to those in GC. However, both microwell culture conditions showed a significant decline in blastocyst formation rates compared to GC culture. This could be explained by the interrupted culture in MG and MS compared to a continuous culture in GC. Transfer in the microwell devices at day 3 involved a change in medium leading to the depletion of putative embryotrophic factors accumulated during the first 3 days of development and an additional manipulation at a time close to compaction, which reportedly represents a critical stage of embryo development [55,56,57,58]. Blastocyst mitochondrial activity and lipid content were assessed as additional markers of developmental competence [36,59]. No significant differences were detected among the three culture conditions in terms of mitochondrial activity, lipid content (per blastocyst area), and the number of lipid droplets per blastocyst. However, a higher prevalence of smaller-sized LDs was revealed in the MS condition. Lipids provide an energy reserve during the initial stages of embryo development before the activation of the embryonic genome and play a key role in plasma membrane biosynthesis [59]. However, excessive lipid droplet formation and increased droplets size, due to perturbed lipid metabolism and mitochondrial function, impair embryo developmental competence and cryotolerance [59,60,61,62]. During morphogenesis, lipid droplets accumulate and grow in size until the morula stage and decrease at the blastocyst stage due to an increased lipid demand [63,64]. The enhanced lipid accumulation in embryos is a consequence of the stress experienced during the in vitro culture procedures [59]. Although no differences in terms of lipid area and LDs number per blastocyst among the three culture conditions were found, it can be hypothesized that the reduced size of lipid droplets in MS blastocysts might be attributed to a higher metabolic activity (and possibly competence) of individually cultured embryos. Consistent with this hypothesis, lipid droplets >6 µm in diameter have been associated with the presence of immature mitochondria and reduced developmental competence [64,65]. Although the blastocyst mean droplets diameter was <6 µm across the three culture conditions, a significantly lower diameter was present in MS compared with MG blastocysts. Further studies are needed to clarify whether individually cultured blastocysts have an enhanced lipid metabolism and cryotolerance compared with embryos cultured in groups. 

## 5. Conclusions

One of main goals in ART is the establishment of an effective individual culture to select euploid high-quality embryos for transfer. The demonstration that individual bovine zygotes can be successfully cultured until the blastocyst stage in ~70 nL of medium would open the way to successfully reach this goal through the application of time-lapse technology coupled to omics analysis on spent media [37,66,67]. Although currently such a nanoliter volume is poorly compatible with the analytical techniques for the detection of single embryo cell free DNA, metabolites, secreted proteins, microRNA, etc., the exponential progresses in this field could allow for the manipulation and analysis of pico- to nanoliter volumes of spent media in the near future. The finding that a bovine embryo can be effectively cultured in approximately 70 nL of medium in a microwell geometry where excessive detrimental solute exchanges between the oil and medium are prevented underscores the importance of extreme confinement in optimizing individual embryo culture. This paves the way for establishing confined individual cultures in larger microwells, which are more conducive to the collection and analysis of spent media.

## Data Availability

Data are freely available by the authors.
